# Measurement of thermodiffusion in liquid ${\textrm{Ag}}_{60}{\textrm{Cu}}_{40}$

**DOI:** 10.1140/epje/s10189-026-00647-y

**Published:** 2026-10-20

**Authors:** Asbjørn Torsvik Krüger, Elke Sondermann, Andreas Meyer

**Affiliations:** Institute for Frontier Materials on Earth and in Space, German Aerospace Center (DLR), Linder Höhe, 51147 Cologne, Germany

## Abstract

**Abstract:**

Thermodiffusion, or the Soret effect, designates a diffusive motion of particles driven by a temperature gradient, leading to a build-up of a concentration gradient. This effect is here measured in the liquid state on the eutectic alloy $\textrm{Ag}_{60}\textrm{Cu}_{40}$, which shows negligible excess volume. No significant thermodiffusion was observed using both in situ X-ray radiography or ex situ energy-dispersive X-ray spectroscopy on separately solidified parts of the sample. This is in contrast to the significant Soret coefficients observed in molten Ag–Al and Al–Ni. Molecular dynamics simulations of thermodiffusion in liquid Ag–Cu [Sarder et al., Philos. Mag. **99**, 468–491 (2019)] report significantly stronger thermodiffusion. However, the trend that thermodiffusion in liquid Ag–Cu is weaker than in other liquid alloys like Al–Ni is supported by our experiment.

**Graphical abstract:**

**Figure:**
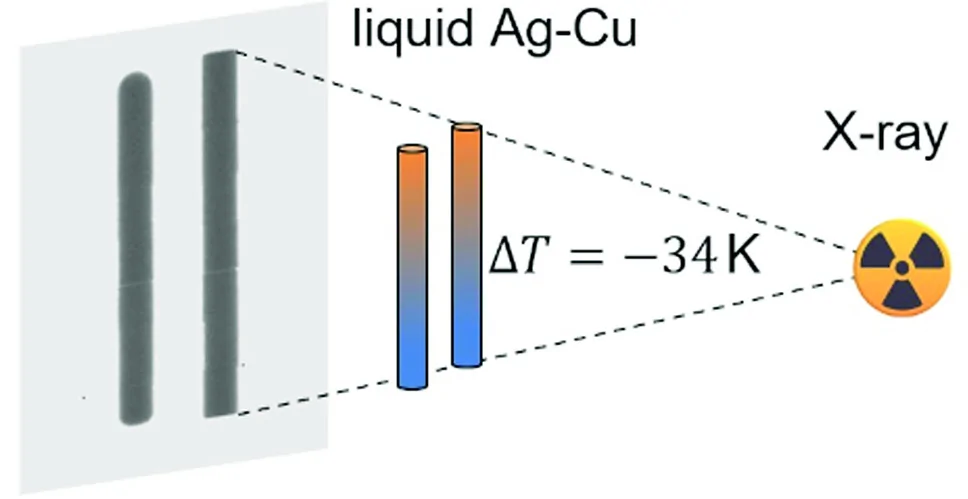

## Introduction

When there is a temperature gradient along a mixture, the different components of the mixture can enrich towards either the hot or cold side [1]. This effect, called the Soret effect after one of its discoverers, influences all kinds of matter, from gases, liquids, to solids [2]. In metals, thermodiffusion can affect solders and the manufacturing of integrated circuits; it has been employed for crystal growth and has been shown to be of use in creating metallic nanowires [3–7].

We have earlier demonstrated that in situ X-ray radiography(XRR) allows time- and space-resolved measurements of thermodiffusion in liquid alloys, where bubbles or free surfaces can be directly detected during the experiment [8–10]. This leads to more precise measurements, as disturbances caused by convection can be detected and excluded from analysis.

In this work, thermodiffusion in the liquid binary alloy Ag–Cu will be investigated.

Silver and copper are the elements with the highest electrical conductivity at room temperature [11]. The two components are used together with tin in ternary Sn–Ag–Cu (SAC) alloys for soldering, in efforts to replace toxic lead-based solders [12]. The Ag–Cu liquid alloy is therefore an interesting subject in the analysis of thermodiffusion, as the Soret effect has been shown to be of importance in solders, where the hot liquid solder is in contact with the cold substrate [3].

The thermodiffusion in liquid Ag–Cu was modelled by Sarder et al. [13], using molecular dynamics (MD) simulations in combination with the Green–Kubo formalism. They found silver to migrate to the cold side, with a Soret coefficient of 1.8e-3 K$^{-1}$ for liquid $\mathrm{Ag_{62.5} Cu_{37.5}}$ at a temperature of 1100 K. This is a value comparable to that found in experiments on similar liquid alloys at comparable temperatures [9, 14, 15].

Still, Sarder et al. [13] deem the observed thermodiffusion to be “very small”, based on their earlier obtained results for Al–Ni, where they found a practically concentration-independent reduced heat of transport of 0.97 eV, which in that alloy translates to a Soret coefficient of 2.6e-3 K$^{-1}$ for liquid $\mathrm{Al_{78.5} Ni_{21.5}}$ at a temperature of 1398 K, with nickel migrating to the cold side [16]. We later experimentally investigated the alloy at that composition and temperature, measuring a Soret coefficient of ${(1.5 \pm 0.3 ) \times 10^{-3}}\textrm{K}^{-1}$ (nickel to the cold side) [8].

It is the goal of this work to measure the Soret coefficient of liquid $\textrm{Ag}_{60}\textrm{Cu}_{40}$ at 1100 K and compare the measured value with that one simulated by Sarder et al. [13]. As a cross-coupling effect between heat and mass transfer, thermodiffusion is very sensitive to the interaction potential in molecular dynamic simulations [17]. Measurements can therefore help to refine simulations.

## Theory

In a binary mixture, the mass diffusion flux **J** is driven by the interdiffusion from a concentration gradient $\boldsymbol{\nabla }c$ and the thermodiffusion from a temperature gradient $\boldsymbol{\nabla }T$, given by [18]

1 $$\begin{aligned} \textbf{J} = -\rho D \boldsymbol{\nabla }c - \rho D_\textrm{T}c_0 \left( 1 - c_0\right) \boldsymbol{\nabla }T . \end{aligned}$$

Here, ρ is the mass density, *D* the interdiffusion coefficient, $D_\textrm{T}$ the thermodiffusion coefficient, and $c_0$ the concentration of one of the two species. After the thermal gradient has been applied and the steady state has been reached (i.e. $\textbf{J} = 0$), the interdiffusion and thermodiffusion processes reach an equilibrium defined by

2 $$\begin{aligned} \boldsymbol{\nabla }c = - \frac{D_\textrm{T}}{D} c_0 \left( 1-c_0 \right) \boldsymbol{\nabla }T , \end{aligned}$$

where the ratio between the thermodiffusion and interdiffusion coefficients is called the Soret coefficient ${S_\textrm{T}}\equiv \frac{D_\textrm{T}}{D}$. For a one-dimensional sample in a linear temperature gradient, the total concentration difference across the sample from the total temperature difference ΔT becomes

3 $$\begin{aligned} \Delta c_\infty = - {S_\textrm{T}}c_0 \left( 1-c_0\right) \Delta T \end{aligned}$$

after reaching equilibrium, while the transient concentration difference is given by [19]

4 $$\begin{aligned} \Delta c(t) = \Delta c_\infty \left( 1 - \frac{8}{\pi ^2} \sum _{n = 0}^\infty \frac{e^{ -\left[ 2n+1\right] ^2 t / \theta }}{\left[ 2n+1\right] ^2} \right) , \end{aligned}$$

where $\theta = \frac{L^2}{\pi ^2 D}$ is the characteristic time, and *L* is the sample length.

## Method

**Fig. 1:**
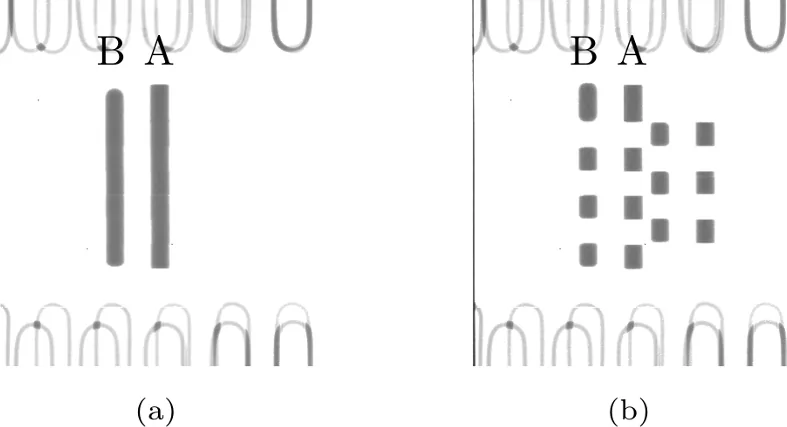
X-ray images from a shear cell experiment, **a** before and **b** after shearing

The samples were alloyed from the pure elements (Ag: Alfa Aesar silver shot 99.99 %, Cu: Alfa Aesar copper rod 99.999 %) in an arc melting furnace under purified argon atmosphere. The rod-shaped samples were inserted into a sample cell which consists of a boron nitride (BN) crucible, sandwiched between heat plates holding heating wires. The temperature at both ends of the samples is measured with thermocouples inside the sample cell. The general setup has been described in an earlier publication of ours [9]. For the experiment presented in this paper, the sample cell was altered so that the sample can be sheared into six segments at the end of the diffusion phase. In this way, the sample concentrations could be measured ex situ as well as in situ, through methods such as energy-dispersive X-ray spectroscopy (EDXS) together with the use of XRR imagery. For X-ray imaging, we used a microfocus X-ray source (XT9160-TED, Viscom AG, Hannover, Germany) and a CdTe detector (XC-Thor series, Direct Conversion AB, Danderyd, Sweden). In this shear cell-type sample cell, the samples are placed in stacked shear plates, with the plates having opposite fixed and movable holes, such that a rod could pass through the plates. At the end point of the thermodiffusive process (at the steady state), the plates were moved, as shown in Fig. 1. The samples are at different depths in the cell, so they do not cross each other during the shearing.

Due to this layering, the shear cell could only host two samples, compared to four in the original furnace cell [9]. After cooling down, the individual samples were extracted from the plates, embedded in epoxy, and analysed in a scanning electron microscope (SEM) with EDXS.

## Results

An experiment with two samples of $\textrm{Ag}_{60}\textrm{Cu}_{40}$ was performed, at a mean temperature of 1099 K.

It should be noted that the in situ XRR identified a free surface in sample B. This can be seen in the X-ray image in Fig. 1 as rounded shape at top and bottom of sample B contrary to the expected flat shape at top and bottom of sample A. A piston mechanism in the sample cell is supposed to adjust the length of the capillary in which the sample rod is placed, so that the capillary is completely filled with molten sample material. For sample B, this mechanism did not function correctly, and the non-wetting behaviour of liquid $\textrm{Ag}_{60}\textrm{Cu}_{40}$ on the BN sample cell resulted in the visible rounded shape.

The free surface in sample B may introduce Marangoni convection, which can significantly alter the observed interdiffusion coefficient, and fully disturb the measured thermodiffusion effect [20, 21].

**Fig. 2:**
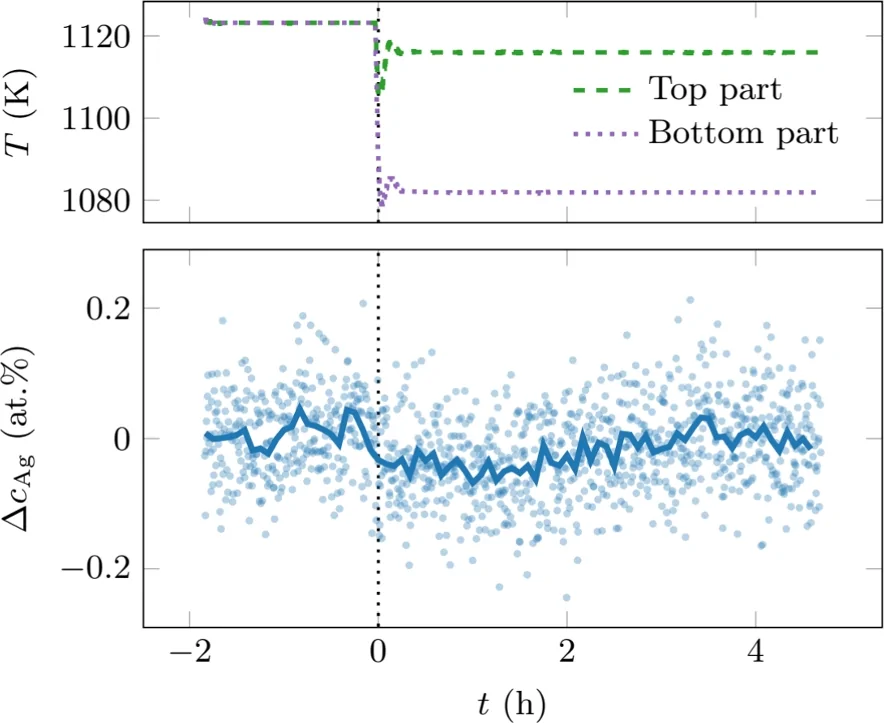
Indicated concentration difference Δc(t) along a sample of $\textrm{Ag}_{60}\textrm{Cu}_{40}$, showing no change. Data from in situ XRR

In Fig. 2 the temperatures at the top and at the bottom of the samples during the experiment are shown. After about two hours of isothermal annealing at 1123 K, a temperature difference of $\Delta T = {-34.2\,\mathrm{\text {K}}}$ around a mean temperature of 1099 K was quickly established. Figure 2 also gives the concentration difference $\Delta c_\textrm{Ag}$ between the top and the bottom part of sample A as function of time as obtained from the XRR imagery. No significant change in the concentration separation was observed.

Due to the weak signal, it was not possible to calculate the interdiffusion coefficient from the transient concentration difference as described in Eq. (4). The Soret coefficient derived from in situ XRR, measured to ${(-0.02 \pm 0.11) \times 10^{-3}\mathrm{K^{-1}}}$, is not significantly different from zero.

**Fig. 3:**
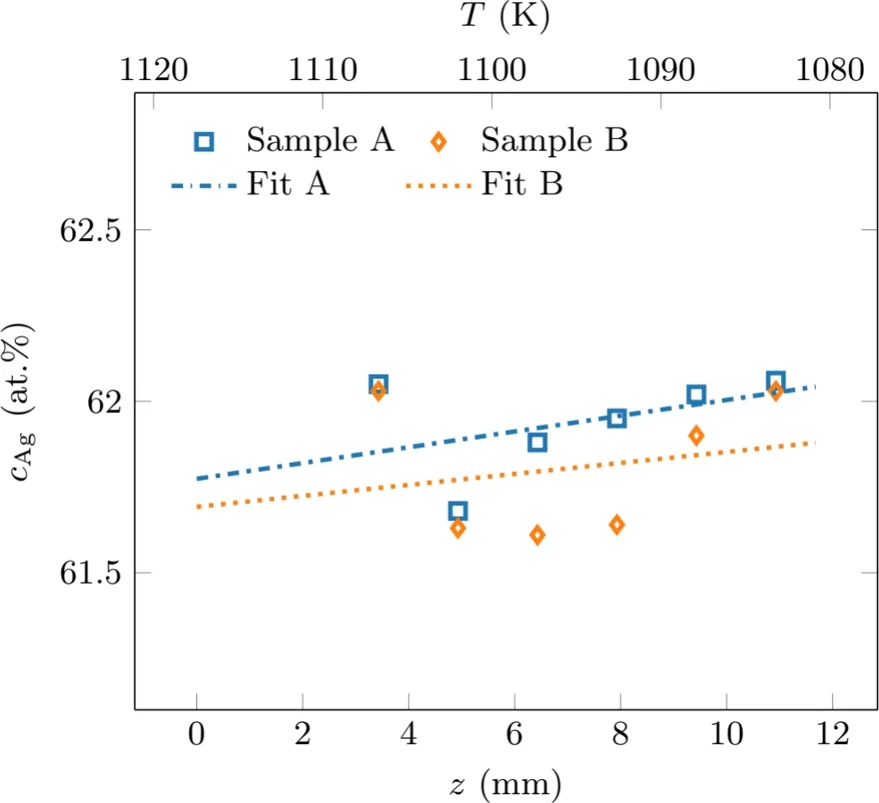
Concentration along two sheared samples of $\textrm{Ag}_{60}\textrm{Cu}_{40}$. Data from ex situ EDXS

After 4.7h with a temperature difference of $\Delta T = {-34.2\,\mathrm{\text {K}}}$, the two liquid $\textrm{Ag}_{60}\textrm{Cu}_{40}$ samples were separated into six pieces each, cooled down, and extracted from the BN plates. The pieces were analysed in a SEM. Using EDXS, the compositions of the different sections were calculated at three different cross sections for each sample piece. The averaged concentration of the six sample pieces is shown in Fig. 3.

The fitted $\frac{\Delta c_\textrm{Ag}}{\Delta T}$ slopes for the two samples with the uncertainty as returned from the fitting function [22], as well as the resulting Soret coefficient ${S_\textrm{T}}$ for the two samples, are given in Table 1.

**Table 1:** Fitted concentration gradient $\frac{\Delta c_\textrm{Ag}}{\Delta T}$ and Soret coefficient ${S_\textrm{T}}$ to the ex situ EDXS data in Fig. 3

Sample	$\frac{\Delta c_\textrm{Ag}}{\Delta T}$ in at.% K$^{-1}$	${S_\textrm{T}}$ in 10$^{-3}$K$^{-1}$
A	-0.007±0.006	0.30±0.27
B	-0.005±0.010	0.21±0.40
A (XRR)	$0.0005\,\, \pm 0.003$	-0.02±0.11

For comparison, the data derived from XRR are also included

As already mentioned, sample B was not properly compressed by the pistons in the sample cell, leading to free surfaces on the top and bottom of that sample, as seen in Fig. 1, possibly generating Marangoni convection disturbing the thermodiffusion process. Sample A had no visible free surfaces, and slightly less uncertainty, but still a very small Soret coefficient. If there is any thermodiffusion in liquid $\textrm{Ag}_{60}\textrm{Cu}_{40}$, it is at least on a much smaller order than other similar liquid alloys, like Ag–Al and Al–Cu [14, 23].

## Discussion

While there is no previously published measurement on thermodiffusion for liquid Ag–Cu, there are results on thermodiffusion for solid Ag–Cu by Jaffe and Shewmon [24], where they presented experiments on trace amounts of radioactive silver in solid copper, measuring a Soret coefficient of ${(-1.5 \pm 0.9) \times 10^{-3}\mathrm{K^{-1}}}$, with silver migrating to the hot side. There have also been investigations on thermodiffusion in solid SAC solder alloys, where Lin et al. [25] investigated the migration of copper between layers of copper, silver and $\mathrm{Ag_{3.2} Sn_{96.8}}$, where they found that the interface of silver and $\mathrm{Ag_{3.2} Sn_{96.8}}$ created a silver-rich interface which reduced the copper migration by a factor of three. However, in earlier measurements of thermodiffusion in both liquid and solid Ag–Al and Al–Cu, there is generally no correlation between the Soret coefficients measured in different phases of a system [9, 14, 26, 27]. Additionally, the measurement in this work was with 60 at. % silver. Compared to the trace amounts of silver in the experiment of Jaffe and Shewmon [24] and the high concentration of tin in the experiment of Lin et al. [25], no conclusions on the relation between these findings can be made.

The interdiffusion of liquid $\textrm{Ag}_{60}\textrm{Cu}_{40}$ was measured by Engelhardt [28], who at 1193 K measured an interdiffusion coefficient of ${(3.7 \pm 0.4) \times 10^{-9}m^{2}s^{-1}}$ with the shear cell technique. With a sample length *L* of 11.0 mm in this experiment, it results in a characteristic time of $\theta \approx {3.3e3\,\mathrm{\text {s}}}$, meaning the experiment ran for a duration of 5.1 characteristic times, and the samples in this experiment achieve more than 99 % of the steady-state concentration separation before shearing. The experiment in this work was performed with a mean temperature of 1099 K, i.e. slightly lower than Engelhardt [28], and therefore also a somewhat lower interdiffusion coefficient is expected. The temperature difference is however small, and it can confidently be assumed that the thermodiffusive process had ample time to establish a concentration separation representative of the steady state.

The lack of a measurable concentration separation after the experiment for liquid $\textrm{Ag}_{60}\textrm{Cu}_{40}$ could either be a indication of a general close-to-zero Soret coefficient, or imply a local minimum of the Soret coefficient, or even a point of reversal of sign of the Soret coefficient, which has been observed to exist for Na–K alloys [29], in addition to in several organic systems [30, 31]. It is also hypothetically possible that at the measured temperature, the Soret coefficient of the liquid $\textrm{Ag}_{60}\textrm{Cu}_{40}$ alloy is negative, which would lead to the denser silver-rich alloy to accumulate at the hot side at the top of the sample column. If the absolute value of such a negative Soret coefficient was large enough, such a setup could overcome the critical Rayleigh number of the system and become unstable, as described by Velarde and Schechter [32]. In that case the unstable density layering would lead to a mixing of the liquid and reduce the detectable concentration gradient. Based on the density data for Ag–Cu from Brillo et al. [33], the Soret coefficient would have to be −5.1e-3 K$^{-1}$ or stronger at 1099 K for an unstable density distribution to occur for liquid $\textrm{Ag}_{60}\textrm{Cu}_{40}$. The build-up of such a large concentration gradient would have been visible in XRR before it became unstable.

As mentioned earlier, the thermodiffusion in liquid Ag–Cu was modelled by Sarder et al. [13], using MD simulations, where they found the Soret coefficient for liquid $\mathrm{Ag_{62.5}Cu{37.5}}$ to be approximately 1.8e-3 K$^{-1}$ at a temperature of 1099 K, with silver migrating to the cold side. This is much larger than what was found in the experiment presented in this work (Table 1) but agrees in the sign of the Soret coefficient from the EDXS data.

The publication of Sarder et al. [13] also simulated other parameters for liquid Ag–Cu, which show similar discrepancy to available experimental data. The model in use (Williams et al. [34]) for example predicts the general shape of the phase diagram for Ag–Cu, but misses the eutectic temperature and concentration by 118 K and 14 at.%, respectively [34, 35]. While the simulated self-diffusion coefficients for pure Ag and Cu are in good agreement with measurements [36, 37], the simulated interdiffusion coefficient is a factor three off a published experiment on the liquid alloy [28]. To summarise, only a qualitative predictability can at most be expected of the Soret coefficient from the simulations of Sarder et al. [13].

The concentration dependency of the Soret coefficient was not experimentally investigated for this work, but the simulated results from Sarder et al. [13] indicate that the coefficient should not change significantly at other concentrations.

### Comparison with predictive models

There have been developed several predictive models for thermodiffusion in binary mixtures, both for organic mixtures and liquid alloys. We here select some of them to compare with the measured result. The selected models were chosen for different reasons: the model of Dougherty and Drickamer [38] has been applied to liquid binary alloys in earlier works [38, 39]. The model of Artola et al. [40] is based on kinetic theory, instead of the non-equilibrium thermodynamic approach of the previously mentioned model, especially interesting with the great amount of investigations into self-diffusion in liquid metals and alloys [37, 41, 42]. The model of Shukla and Firoozabadi [43], another non-equilibrium thermodynamic model, has been found to be the most reliable to predict thermodiffusion in Lennard-Jones (LJ) mixtures [44, 45].

For these models, the following sources were used for the thermophysical properties required as input parameters: for the Gibbs energy, the excess Gibbs energy parameters from Witusiewicz [35] were used. The energy of vaporisation for the elements was sourced from Yaws and Satyro [46], while the densities and activation energies of viscous flow were from Assael et al. [47, 48]. The activation energy of self-diffusion from quasi-elastic neutron scattering by Engelhardt et al. [37] was utilised for silver and similarly by Meyer [41] for copper.

The Soret coefficients for $\textrm{Ag}_{60}\textrm{Cu}_{40}$ as predicted by the models are shown in Table 2, as calculated for a mean temperature of 1099 K, the same as for the experiment.

**Table 2:** Calculated Soret coefficients for liquid $\textrm{Ag}_{60}\textrm{Cu}_{40}$ with different models at a mean temperature of 1099 K

Model	Predicted ${S_\textrm{T}}$
Dougherty and Drickamer [38]	${-4.1 \times 10^{-3}\mathrm{K^{-1}}}$
Shukla and Firoozabadi [43]	${0.1 \times 10^{-3}\mathrm{K^{-1}}}$
Artola et al. [40]	${-1.1 \times 10^{-3}\mathrm{K^{-1}}}$

Only the model of Shukla and Firoozabadi [43] is within the uncertainty of the measurement itself. As the measured Soret coefficient is so close to zero, it cannot be clearly established whether the models predict the incorrect sign, but it is at least clear that the absolute value of the Soret coefficient is misrepresented by several of the models. It should be noted that the model of Shukla and Firoozabadi [43] also predicts a close-to-zero Soret coefficient for other alloys where the Soret coefficient is decisively nonzero in measurements, such as for liquid Ag–Al and Al–In [9, 10, 49].

Soret coefficients recently measured in liquid alloys Ag–Al [9], Al–Ni [8], or Al–In [10] are found to be within an order of magnitude. The measurement of liquid $\textrm{Ag}_{60}\textrm{Cu}_{40}$ presented here is an exception, giving a value around zero. Of the mentioned alloys, liquid Ag–Cu is the only system with a negligible excess volume determined from density measurements [50]. This could hint to the thermodiffusion being dependent on the excess volume, as has been theorised by Morozov [51]. Future studies will show whether this dependence can be confirmed.

## Conclusion

For liquid Ag–Cu, at most a barely significant migration of silver towards the cold side was observed, using an ex situ analysis via EDXS of the processed samples. The Soret coefficient derived from in situ XRR, measured to ${(-0.02 \pm 0.11) \times 10^{-3} K^{-1}}$, is not significantly different from zero. In previously published MD simulations, the migration of silver towards the cold side was observed, with a higher Soret coefficient than that found in the present measurements. From the comparison of the other parameters of the simulations (e.g. eutectic point) with earlier measurements on those parameters, it is argued that only a qualitative match in such a sensitive cross-effect as thermodiffusion can be expected. The weakness of the Soret coefficient in $\textrm{Ag}_{60}\textrm{Cu}_{40}$ compared to other liquid alloys may hint at a dependency of thermodiffusion on the excess volume.

## Data Availability

X-ray images from the presented experiment are available from the corresponding author on reasonable request.
